# Auxiliary KCNE subunits modulate both homotetrameric Kv2.1 and heterotetrameric Kv2.1/Kv6.4 channels

**DOI:** 10.1038/srep12813

**Published:** 2015-08-05

**Authors:** Jens-Peter David, Jeroen I. Stas, Nicole Schmitt, Elke Bocksteins

**Affiliations:** 1 Danish National Research Foundation Centre for Cardiac Arrhythmia and Department for Biomedical Sciences, Faculty of Health and Medical Sciences, University of Copenhagen, Copenhagen, Denmark; 2 Laboratory for Molecular Biophysics, Physiology and Pharmacology, Department for Biomedical Sciences, University of Antwerp, Antwerp, Belgium

## Abstract

The diversity of the voltage-gated K^+^ (Kv) channel subfamily Kv2 is increased by interactions with auxiliary β-subunits and by assembly with members of the modulatory so-called silent Kv subfamilies (Kv5-Kv6 and Kv8-Kv9). However, it has not yet been investigated whether these two types of modulating subunits can associate within and modify a single channel complex simultaneously. Here, we demonstrate that the transmembrane β-subunit KCNE5 modifies the Kv2.1/Kv6.4 current extensively, whereas KCNE2 and KCNE4 only exert minor effects. Co-expression of KCNE5 with Kv2.1 and Kv6.4 did not alter the Kv2.1/Kv6.4 current density but modulated the biophysical properties significantly; KCNE5 accelerated the activation, slowed the deactivation and steepened the slope of the voltage-dependence of the Kv2.1/Kv6.4 inactivation by accelerating recovery of the closed-state inactivation. In contrast, KCNE5 reduced the current density ~2-fold without affecting the biophysical properties of Kv2.1 homotetramers. Co-localization of Kv2.1, Kv6.4 and KCNE5 was demonstrated with immunocytochemistry and formation of Kv2.1/Kv6.4/KCNE5 and Kv2.1/KCNE5 complexes was confirmed by Fluorescence Resonance Energy Transfer experiments performed in HEK293 cells. These results suggest that a triple complex consisting of Kv2.1, Kv6.4 and KCNE5 subunits can be formed. *In vivo*, formation of such tripartite Kv2.1/Kv6.4/KCNE5 channel complexes might contribute to tissue-specific fine-tuning of excitability.

Voltage-gated K^+^ (Kv) channels are tetrameric transmembrane proteins that open and close in response to changes in the membrane potential. An assembled Kv channel contains a central K^+^ conducting pore formed by the S5-S6 segments of each α-subunit, four voltage-sensing domains (VSDs) generated by the different S1-S4 segments and cytoplasmic N- and C-termini^1^. Remarkably and in contrast to Kv1, Kv3 and Kv4 subfamilies, Kv2 channels assemble with modulatory α-subunits of Kv5, Kv6, Kv8 and Kv9 subfamilies. These channel proteins are designated silent Kv (KvS) subunits since they fail to form functional channels at the plasma membrane when expressed alone, despite possessing most of the hallmarks of a typical Kv subunit. Upon co-assembly with Kv2 subunits, KvS subunits change the voltage-dependence of both activation and inactivation, activation and deactivation kinetics and/or the current density compared to Kv2 homotetramers (for review, see^2^).

In addition, cytoplasmic and/or transmembrane auxiliary β-subunits can associate with a functional Kv2 channel, thereby increasing the diversity of the channel by modulating channel expression and its biophysical properties. For Kv2.1, several auxiliary subunits have been described. The cytoplasmic protein KChAP acts as a chaperon protein and increases the amount of functional Kv2.1 channels at the plasma membrane^3^. The transmembrane proteins AMIGO and KCNE1-3 mainly affect biophysical properties; AMIGO increases the Kv2.1 current density by shifting the voltage-dependence of activation to hyperpolarized potentials by 10 mV^4^, whereas KCNE1, KCNE2, and KCNE3 slow activation kinetics^5^,^6^,^7^.

The KCNE family consists of five members, KCNE1-5 (also known as MinK (KCNE1) and MinK-related proteins (MiRP) 1–4 (KCNE2-5)). They harbor a single transmembrane α-helix, an extracellular N-terminus and an intracellular C-terminus. All members interact with a wide variety of ion channel α-subunits (for review, see^8^), where the best studied interaction is the modulation of Kv7.1 by KCNE1 to obtain a current similar to the cardiac *I*_Ks_-current^9^,^10^. KCNE1-5 subunits are expressed in a wide range of mammalian tissues. While the role of KCNE1-3 is well described in cardiac, pancreatic or intestinal physiology, the physiological functions of KCNE4-5 are less understood (for review, see^8^).

It has been suggested that different auxiliary β-subunits can associate simultaneously within Kv channel complexes. For example, KChAP and Kvβ1.2 proteins can be simultaneously present in Kv1.4, Kv1.5, Kv2.1 and Kv4.3-containing channels^11^ while KCNE1 and KCNE4 might co-associate with Kv7.1 channels into a “triple subunit” channel complex^12^,^13^. In this study, we sought to investigate whether auxiliary β-subunits and modulatory α-subunits can simultaneously associate with Kv α-subunits into functional tripartite channel complexes. To this end, we examined the effect of co-expressing human Kv2.1 and the modulatory α-subunit Kv6.4 with auxiliary β-subunits of the KCNE family.

## Results

### KCNE1, KCNE2, and KCNE3 moderately affect Kv2.1 and Kv2.1/Kv6.4 channels

Earlier studies provided evidence that KCNE1-3 interact with rat Kv2.1 subunits resulting in channels that possess altered biophysical properties and changed current densities^5^,^6^. However, these KCNE-induced effects depended on the KCNE species; e.g. rat KCNE1 slowed Kv2.1 activation ~2-fold and reduced the Kv2.1 current density, while human KCNE1 did only alter Kv2.1 activation significantly at +60 mV and did not affect the Kv2.1 current density significantly^6^. Therefore, we first tested the effect of human KCNE1-3 on human Kv2.1 channels. Co-expression of Kv2.1 with KCNE1 or KCNE3 reduced the current density significantly and slowed Kv2.1 activation slightly (although significantly at +60 mV) without changing the voltage-dependences of activation or inactivation or the deactivation kinetics. In case of KCNE2, only the current density was reduced (Suppl. Fig. S1, Suppl. Table S1).

Next, we tested whether co-expression of KCNE1-3 subunits affected human Kv2.1/Kv6.4 heterotetrameric channels. Compared to Kv2.1 homotetramers, Kv2.1/Kv6.4 heterotetramers display two components in their activation kinetics and an approximately 40 mV shifted voltage-dependence of inactivation into hyperpolarized direction compared to Kv2.1 homotetramers^14^. In contrast to their effect on Kv2.1 homotetramers, KCNE1 and KCNE3 did not affect the activation kinetics of Kv2.1/Kv6.4 heterotetramers, while KCNE2 accelerated the fast component of the Kv2.1/Kv6.4 activation slightly (significantly at +60 mV) (Suppl. Fig. S2, Suppl. Table S1). Co-expression of the KCNE1-3 subunits did not alter the current density or voltage-dependence of activation or inactivation of Kv2.1/Kv6.4 heterotetramers. In addition, the deactivation kinetics of Kv2.1/Kv6.4 heterotetramers were unchanged by KCNE1-3 subunits (Suppl. Fig. S2, Suppl. Table S1). Our results support previous findings that KCNE1-3 interact with Kv2.1^5^,^6^.

### KCNE4 modulates both Kv2.1 and Kv2.1/Kv6.4 channel complexes

An interaction of KCNE4-5 with Kv2.1 has not yet been investigated. However, Kv2.1, KCNE4 and KCNE5 subunits can all be detected in the heart and the brain^8^,^15^,^16^ creating the possibility that KCNE4-5 subunits affect Kv2.1 homotetramers *in vivo*. In addition, Kv6.4 has been found expressed in the brain^14^. Therefore, we investigated the effect of KCNE4-5 on both Kv2.1 and Kv2.1/Kv6.4 channels.

**Figure 1:**
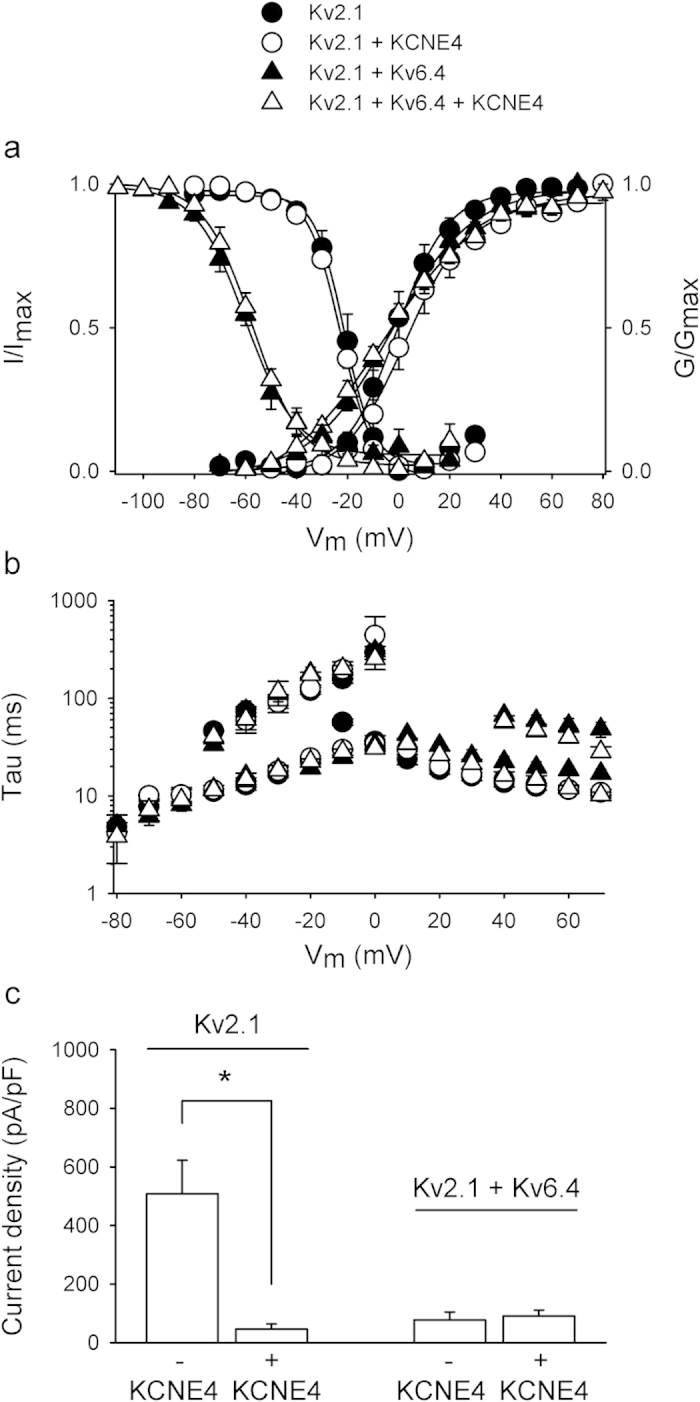
KCNE4 modulates both Kv2.1 homotetramers and Kv2.1/Kv6.4 heterotetramers. (**A)** Voltage-dependence of activation and inactivation of Kv2.1 and Kv2.1/Kv6.4 in the absence or presence of KCNE4. The activation curve was determined by plotting the normalized tail currents at −35 mV as a function of the prepulse potential and the inactivation curve was determined by plotting the normalized current amplitude at +60 mV as a function of the 5-s prepulse potential. Solid lines represent the Boltzmann fit. KCNE4 (open symbols) did not modulate Kv2.1 (circles) or Kv2.1/Kv6.4 (triangles) voltage-dependence of activation and inactivation. (**B)** Activation and deactivation kinetics of Kv2.1 and Kv2.1/Kv6.4 in the absence or presence of KCNE4 derived from a single or double exponential fit of the raw current recordings. KCNE4 did not affect Kv2.1 activation or deactivation kinetics significantly whereas it slightly fastened Kv2.1/Kv6.4 activation kinetics at higher potentials. (**C)** Current densities obtained at 0 mV after co-expression of 250 ng Kv2.1 + 1 μg CFP or 1 μg KCNE4 (1^st^ and 2^nd^ combination, respectively) and 0.5 μg Kv2.1 and 5 μg Kv6.4 + 1 μg CFP or 1 μg KCNE4 (3^rd^ and 4^th^ combination, respectively). Co-expression of KCNE4 reduced Kv2.1 current density significantly (*p < 0.05) while co-expression of KCNE4 with Kv2.1/Kv6.4 had no significant effect.

Co-expression of KCNE4 did not affect the biophysical properties of Kv2.1 homotetramers (Fig. 1A,B, Table 1) but did reduce the Kv2.1 current density ~10-fold (508 ± 114 pA/pF, n = 21 and 46 ± 18, n = 5 in the absence and presence of KCNE4, respectively, Fig. 1C). Compared to Kv2.1 homotetramers, Kv2.1/Kv6.4 heterotetramers display a significantly reduced current density^17^ yet co-expression of KCNE4 did not reduce the Kv2.1/Kv6.4 current density further. In contrast, current density was slightly, yet not significantly increased (77 ± 27 pA/pF, n = 12 vs. with KCNE4 91 ± 20 pA/pF, n = 7, Fig. 1C). In addition, co-expression of KCNE4 modulated the biophysical Kv2.1/Kv6.4 properties slightly. KCNE4 accelerated the fast component of Kv2.1/Kv6.4 activation significantly at higher potentials (p < 0.05) but did not affect the slow Kv2.1/Kv6.4 activation component, the deactivation kinetics, or the voltage-dependencies of activation and inactivation (Fig. 1A,B, Table 1).

### KCNE5 modulates both Kv2.1 homotetramers and Kv2.1/Kv6.4 heterotetramers

**Figure 2:**
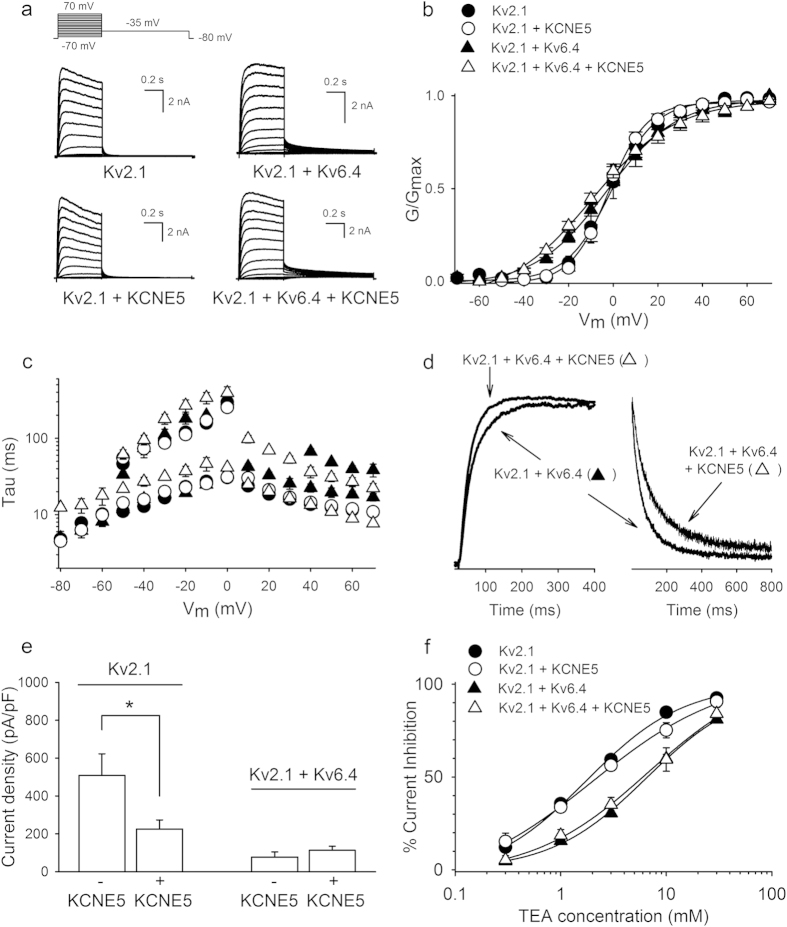
KCNE5 modulates both Kv2.1 homotetramers and Kv2.1/Kv6.4 heterotetramers. (**A)** Representative current recordings to determine the Kv2.1 (left) and Kv2.1/Kv6.4 (right) activation properties in the absence (top) and presence (bottom) of KCNE5. The applied pulse protocol is given on top. (**B)** Voltage-dependence of activation of Kv2.1 and Kv2.1/Kv6.4 in the absence or presence of KCNE5. The activation curve was obtained as in Supplemental Figs 1, 2, 3. KCNE5 (open symbols) did not modulate Kv2.1 (circles) or Kv2.1/Kv6.4 (triangles) voltage-dependence of activation. (**C)** Kv2.1 and Kv2.1/Kv6.4 activation and deactivation kinetics in the absence or presence of KCNE5. KCNE5 had no significant effect on Kv2.1 kinetics but accelerated and slowed the fast component of Kv2.1/Kv6.4 channel activation and deactivation, respectively. (**D)** Scaled activation (at +20 mV, left) and deactivation (at -30 mV, right) current traces of Kv2.1/Kv6.4 in the absence or presence of KCNE5. (**E)** Current densities obtained at 0 mV after co-expression of 250 ng Kv2.1 + 1 μg CFP (1^st^ combination), 250 ng Kv2.1 + 1 μg KCNE5 (2^nd^ combination), 0.5 μg Kv2.1 + 1 μg CFP + 5 μg Kv6.4 (3^rd^ combination) and 0.5 μg Kv2.1 + 1 μg KCNE5 + 5 μg Kv6.4 (4^th^ combination). Co-expression of KCNE5 with Kv2.1 (left) resulted in a ~2-fold reduction of the current density (*p<0.05) while co-expression of KCNE5 with Kv2.1/Kv6.4 had no significant effect. (**F)** TEA concentration-effect curves of Kv2.1 and Kv2.1/Kv6.4 in the absence or presence of KCNE5. Concentration-effect curves were obtained by plotting the normalized percentage of current inhibition at +30 mV as a function of the applied TEA concentration and the Hill-equation was fitted to the data. KCNE5 did not significantly alter the TEA sensitivity of either Kv2.1 or Kv2.1/Kv6.4 channels.

Typical current recordings of Kv2.1 and Kv2.1/Kv6.4 channels in the absence and presence of KCNE5 are shown in Fig. 2A. Co-expression of KCNE5 with Kv2.1 alone did neither alter the voltage-dependence of activation (Fig. 2B, Table 1) nor activation nor deactivation kinetics (Fig. 2C, Table 1). Yet, co-expression of KCNE5 with Kv2.1 and Kv6.4 resulted in modified activation and deactivations kinetics (Fig. 2C,D, Table 1). The scaled current traces in Fig. 2D represent Kv2.1/Kv6.4 and Kv2.1/Kv6.4/KCNE5 activation and deactivation at +20 mV and −30 mV, respectively. KCNE5 accelerated the fast component of Kv2.1/Kv6.4 activation ~2-fold between +20 mV and +70 mV without affecting the slow Kv2.1/Kv6.4 activation component (Fig. 2C, Table 1). With respect to deactivation, KCNE5 decreased the fast component ~2- (at −10 mV) to ~3-fold (at −80 mV) without affecting the slow component of Kv2.1/Kv6.4 deactivation significantly (Fig. 2C, Table 1).

Notably, co-expression of KCNE5 did not influence the biophysical properties of Kv2.1, but affected Kv2.1 current density significantly; KCNE5 decreased Kv2.1 current density ~ 2-fold from 508 ± 114 pA/pF (n = 21) in the absence of KCNE5 to 225 ± 48 pA/pF (n = 23) in the presence of KCNE5 (Fig. 2E). Yet, KCNE5 modulated Kv2.1/Kv6.4 biophysical properties significantly but did not decrease Kv2.1/Kv6.4 current density further (Fig. 2E). KCNE5 even tended to increase the Kv2.1/Kv6.4 current density (77 ± 27 pA/pF, n = 12 and 113 ± 21 pA/pF, n = 17 in the absence and presence of KCNE5, respectively), although not reaching significance, which might be expected by accelerated activation and decreased deactivation rates.

In addition to their effect on channel gating and current density of Kv channels, KCNE subunits can also modify the pharmacological profile (for review, see^8^). It has been demonstrated earlier that KCNE2 subunits altered the sensitivity of Kv2.1 to the common channel pore blocker tetraethylammonium (TEA)^5^. Hence, we determined the effect of TEA on Kv2.1 and Kv2.1/Kv6.4 in the absence and presence of KCNE5 (Fig. 2F). The concentration-effect curve showed no modification of IC_50_ values for TEA of Kv2.1 and Kv2.1/Kv6.4 channels by KCNE5 (1.7 ± 0.4 mM and 1.8 ± 0.3 mM for Kv2.1 homotetramers and 7.6 ± 1.0 mM and 8.3 ± 1.8 mM for Kv2.1/Kv6.4 heterotetramers in the absence or presence of KCNE5, respectively).

**Figure 3:**
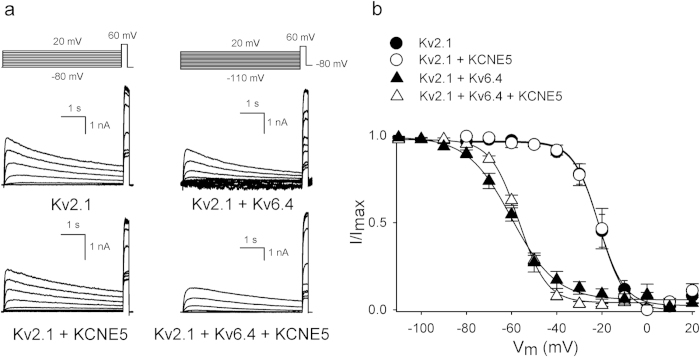
KCNE5 modulates the voltage-dependence of inactivation of Kv2.1/Kv6.4 heterotetramers. (**A).** Representative current recordings to determine the voltage-dependence of inactivation of Kv2.1 homotetramers (left) and Kv2.1/Kv6.4 heterotetramers (right) in the absence (top) or presence (bottom) of KCNE5. The applied voltage protocols are illustrated on top of the respective current tracings. (**B).** Voltage-dependence of inactivation of Kv2.1 and Kv2.1/Kv6.4 in the absence or presence of KCNE5. The inactivation curve was obtained as in Supplemental Fig. 1. KCNE5 (open symbols) did not alter the voltage-dependence of inactivation of Kv2.1 (circles) but steepened the slope of the Kv2.1/Kv6. inactivation curve (triangles) significantly (p<0.05).

**Figure 4:**
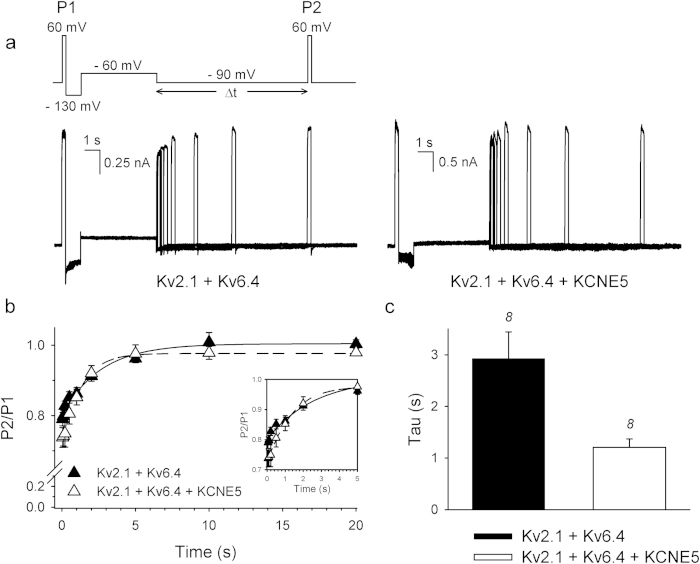
KCNE5 accelerates the recovery of Kv2.1/Kv6.4 closed-state inactivation. (**A)** Representative current recordings in the absence (left) and presence (right) of KCNE5 obtained from the pulse protocol shown on top to investigate the recovery of Kv2.1/Kv6.4 closed-state inactivation. (**B)** Recovery of Kv2.1/Kv6.4 closed-state inactivation in the absence (filled symbols) or presence (open symbols) of KCNE5 obtained by plotting the normalized P2/P1 current amplitudes as function of the pulse duration at the −90 mV voltage step. Single exponential functions were fitted to the Kv2.1/Kv6.4 recovery in absence or presence of KCNE5 (solid and dotted line, respectively). Inset represents a close-up of the initial phase of the recovery of closed-state inactivation. **(C)** Recovery time constants of the closed-state inactivation of Kv2.1/Kv6.4 in the absence (black) or presence of KCNE5 (white). KCNE5 accelerated the recovery of Kv2.1/Kv6.4 closed-state inactivation significantly (*p < 0.05); n=number of cells.

Since Kv6.4 subunits shift the voltage-dependence of inactivation of Kv2.1/Kv6.4 heterotetramers ~40 mV into hyperpolarized direction compared to that of Kv2.1 homotetramers^14^, we investigated the effect of KCNE5 on Kv2.1 and Kv2.1/Kv6.4 inactivation properties (Fig. 3). Figure 3A shows typical current recordings to determine the voltage-dependence of inactivation of Kv2.1 and Kv2.1/Kv6.4 channels in the absence and presence of KCNE5. Co-expression of KCNE5 with Kv2.1 did not alter the voltage-dependence of Kv2.1 channels (Fig. 3B, Table 1). However, when co-expressed with Kv2.1 and Kv6.4 subunits, KCNE5 significantly altered the voltage-dependency of Kv2.1/Kv6.4 inactivation, rendering the inactivation curve steeper without altering the midpoint significantly (Fig. 3B, Table 1). The slope of the inactivation curve depends on the ratio of initiation and recovery of the Kv6.4-induced closed-state inactivation. Therefore, we determined both the initiation and recovery rate constant of Kv2.1/Kv6.4 closed-state inactivation in the absence and presence of KCNE5 (Fig. 4). The recovery rate was determined using the protocol illustrated in Fig. 4A: an initial control pulse to +60 mV (P1) was used to record the initial current amplitude, followed by a 1 s step to −130 mV to recover all channels from inactivation, then a 10 s pulse to −60 mV to induce a certain degree of closed-state inactivation, a pulse of variable duration to −90 mV allowing the channels to recover from inactivation and a second test pulse to +60 mV (P2). The fraction P2/P1 represents the degree of channels that have recovered from their closed-inactivated state and was plotted as function of time spent at the recovery pulse to −90 mV (Fig. 4B). Kv2.1/Kv6.4 channels recovered with a time constant of 2.9 ± 0.5 s (n = 8) and KCNE5 significantly modulated this process, increasing the rate of recovery to 1.2 ± 0.2 s (Fig. 4C). In addition, the rate of initiation of Kv2.1/Kv6.4 closed-state inactivation and the extent of inactivation was determined in the absence and presence of KCNE5 but no significant differences could be observed (data not shown).

**Figure 5:**
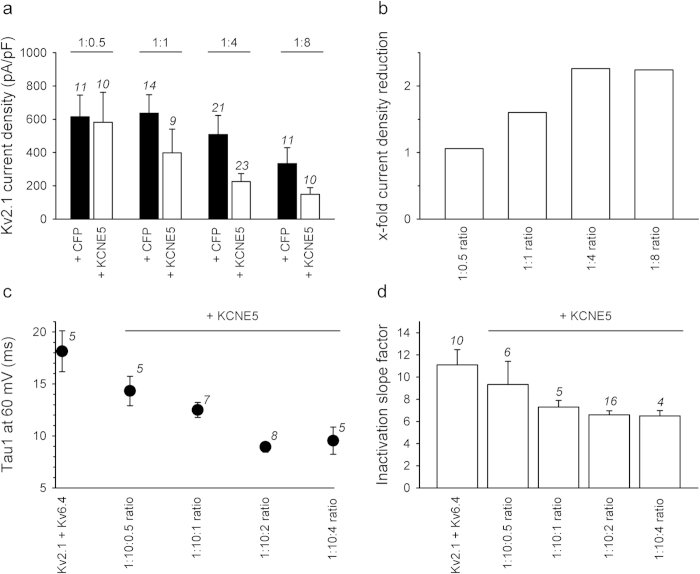
Increased KCNE5 concentrations result in increased modulations of Kv2.1 homotetramers and Kv2.1/Kv6.4 heterotetramers. (**A)** Current densities determined at 0 mV after co-expression of Kv2.1 with CFP (black) or KCNE5 (white) in a 1:0.5 (250 ng + 125 ng), 1:1 (250 ng + 250 ng), 1:4 (250 ng + 1 μg) and 1:8 (250 ng + 2 μg) cDNA transfection ratio. (**B)** Relative KCNE5-induced reduction of the Kv2.1 current density determined by dividing the Kv2.1 current density obtained upon co-expression with CFP by that upon co-expression with KCNE5 (black and white bars in panel A, respectively). The different transfection cDNA ratios are the same as in panel A. (**C)** Fast activation component (at 60 mV) of 0.5 μg Kv2.1 + 5 μg Kv6.4 (1:10 transfection ratio) in the absence or presence of 250 ng, 0.5 μg, 1 μg or 2 μg KCNE5 (1:10:0.5, 1:10:1, 1:10:2 and 1:10:4 cDNA transfection ratios, respectively). (**D)** Slope factor of the voltage-dependence of inactivation of Kv2.1/Kv6.4 in the absence or presence of KCNE5 transfected in different cDNA ratios. The used cDNA transfection ratios are the same as in panel C. In each panel, the numbers in italic represent the number of cells.

Next, we determined the impact of different levels of KCNE5 expression on Kv2.1 and Kv2.1/Kv6.4 channels (Fig. 5). We co-transfected Kv2.1 or Kv2.1/Kv6.4 with KCNE5 in different ratios and determined the impact of KCNE5 focusing on those properties that we had seen to be mostly influenced by KCNE5 co-expression, i.e. Kv2.1 current density (Fig. 5A,B), and Kv2.1/Kv6.4 activation rate (Fig. 5C) and voltage-dependence of inactivation (Fig. 5D). Co-expression of Kv2.1 with KCNE5 in a 1:0.5, 1:1, 1:4 and 1:8 (Kv2.1:KCNE5) cDNA ratio reduced Kv2.1 current densities gradually. Although Kv2.1 current densities also decreased with increasing amounts of transfected peCFP vector alone (used to correct for effects of cDNA dilution and/or overload of the transcriptional/translational machinery), we observed a clear KCNE5-mediated decrease in Kv2.1 current density (Fig. 5A,B). These results demonstrated that at least a part of the Kv2.1 current reduction observed upon co-expression with KCNE5 is caused by KCNE5 and this KCNE5-induced current reduction depends on the KCNE5 expression level. Similarly, co-expression of Kv2.1/Kv6.4 channels (obtained by a 1:10 Kv2.1:Kv6.4 cDNA transfection ratio to minimize the presence of Kv2.1 homotetramers) with KCNE5 in 1:10:0.5, 1:10:1, 1:10:2 and 1:10:4 (Kv2.1:Kv6.4:KCNE5) cDNA ratios revealed that KCNE5 gradually accelerated the activation kinetics and steepened the voltage-dependence of inactivation (Fig. 5C,D).

### Kv2.1, Kv6.4 and KCNE5 subunits co-localize in HEK293 cells

**Figure 6:**
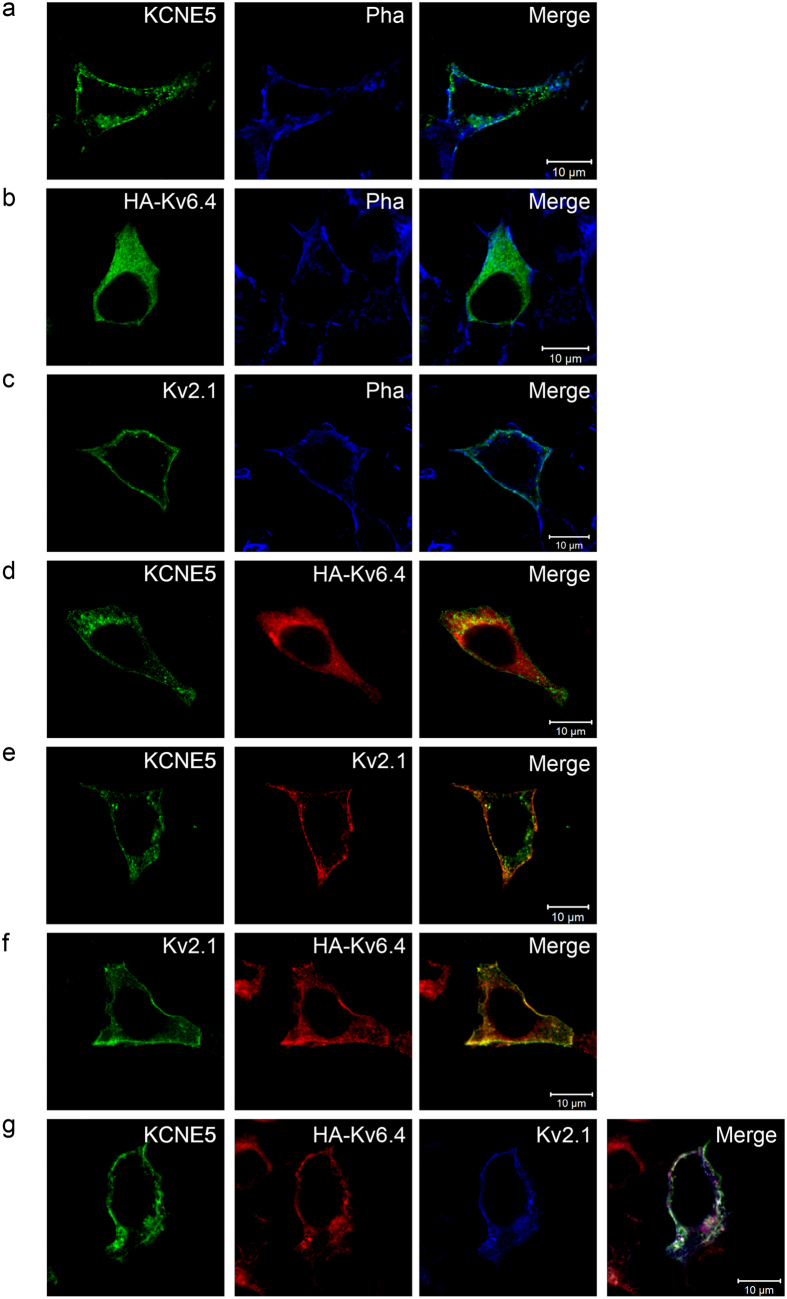
KCNE5 co-localizes with both Kv2.1 homotetramers and Kv2.1/Kv6.4 heterotetramers. (**A–C)** Horizontal confocal images of HEK293 cells singly expressing KCNE5, HA-Kv6.4, and Kv2.1, respectively. Both KCNE5 and Kv2.1 were found in clusters at the plasma membrane, whereas HA-Kv6.4 was retained intracellularly. Phalloidin (Pha) that labels the submembranous actin cytoskeleton was used to visualize plasma membrane localization. (**D)** Co-expressing KCNE5 and HA-Kv6.4 did not result in co-localization of the subunits. Hence, KCNE5 was unable to rescue HA-Kv6.4 to the cell surface. (**E–F)** Co-expressing Kv2.1 and KCNE5 or HA-Kv6.4 resulted in partial overlap of the subunits in small clusters at the cell membrane. Thus, Kv2.1 is capable of redistributing the HA-Kv6.4 subunit to the cell surface and potentially form complexes with both KCNE5 and HA-Kv6.4, respectively. (**G)** Co-localization of all three subunits was detected when they were transiently expressed in the same cells, which indicates the possibility of a triple complex formation. Merged images are shown in the right column of each row.

Since the modulation of Kv2.1 and Kv2.1/Kv6.4 channels by KCNE5 subunits has previously not been demonstrated and co-expression of KCNE5 modified Kv2.1/Kv6.4 currents most extensively (compared to the other KCNE subunits), we assessed whether KCNE5 associates with Kv2.1 and Kv6.4 subunits into Kv2.1/KCNE5 and Kv2.1/Kv6.4/KCNE5 channel complexes in HEK293 cells (Fig. 6). We performed immunocytochemical experiments and found Kv2.1 subunits in small clusters at the cell surface when expressed alone in line with previous studies (Fig. 6C)^18^. In addition, Kv6.4 subunits were retained intracellularly (Fig. 6B) which corresponds to the previously demonstrated ER retention of Kv6.4 subunits when expressed alone^14^. As described earlier, this ER retention is relieved upon co-expression with Kv2.1 subunits resulting in Kv2.1/Kv6.4 heterotetramers at the plasma membrane^14^,^19^ which we also observed in our immunocytochemical experiments (Fig. 6F). To examine whether KCNE5 interacts with Kv2.1 and Kv6.4 in (tripartite) channels, we first deduced the KCNE5 localization pattern when expressed alone in HEK293 cells using a custom antibody directed against KCNE5 (Suppl. Material and Methods, Suppl. Fig. S3). We found that KCNE5 was located mainly in small vesicles or clusters which appeared to be at the plasma membrane (Fig. 6A). Performing surface stainings using a N-terminally HA-tagged KCNE5 construct, we found that KCNE5 was able to traffic to the cell membrane on its own and was located in small clusters (Suppl. Fig. S4, arrows) as seen for Kv2.1 (Fig. 6C). From the whole cell stainings it was noticeable that KCNE5 was also located in some vesicles beneath the membrane. To determine the origin of these vesicles/clusters we employed different compartmental markers (Suppl. Fig. S5). These vesicles were not part of the ER, Golgi Apparatus, late endosomes/prelysosomes or early endosomes (Suppl. Fig. S5A-C, E, respectively), yet some were found in vesicles positive for the transferrin receptor, a marker for recycling endosomes. (Suppl. Fig. S5D). Co-expression of KCNE5 and Kv2.1 revealed that the channel subunits located in the same clusters (Fig. 6E). In contrast to Kv2.1, KCNE5 was incapable of rescuing Kv6.4 from the ER (Fig. 6D), yet when all three subunits were co-expressed in the same cell they co-localized at the cell surface (Fig. 6G). To ensure that this observed co-localization was originating from the formed Kv2.1/Kv6.4/KCNE5 channel complexes (rather than from non-specific interactions caused by the presence of the HA tag), we confirmed electrophysiologically that the channel properties were not affected by the HA tag (Suppl. Fig. S6).

In summary, these experiments demonstrate that KCNE5 is able to traffic to the membrane where it co-localizes with both homotetrameric Kv2.1 and heterotetrameric Kv2.1/Kv6.4 channels. Hence, this supports that KCNE5 is capable of assembling with Kv2.1 and Kv6.4 subunits into both functional Kv2.1/KCNE5 and Kv2.1/Kv6.4/KCNE5 channel complexes, respectively.

### Kv2.1, Kv6.4 and KCNE5 subunits physically associate within channel complex

**Figure 7:**
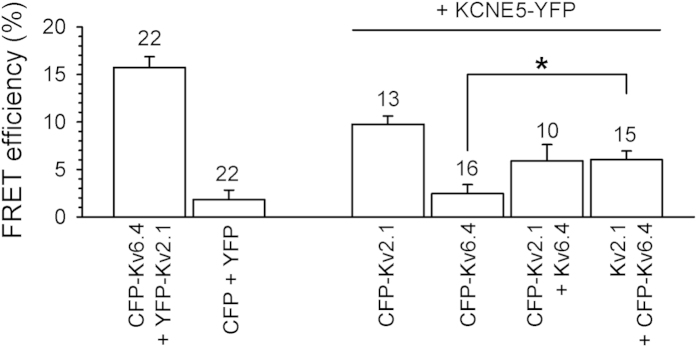
KCNE5 associates with both Kv2.1 homotetramers and Kv2.1/Kv6.4 heterotetramers. Average FRET efficiencies determined after co-expression of CFP- and YFP- labeled subunits. CFP-Kv6.4 + YFP-Kv2.1 and CFP + YFP combinations represent the positive and negative control, respectively. Note the increased FRET efficiency after co-expression of CFP-Kv6.4 with YFP-KCNE5 and untagged Kv2.1 compared to the CFP-Kv6.4 + YFP-KCNE5 co-expression; n = number of cells; *p < 0.05.

To address whether KCNE5 subunits physically associate with Kv2.1 and Kv6.4 within Kv2.1/KCNE5 and Kv2.1/Kv6.4/KCNE5 channel complexes we determined the Fluorescence Resonance Energy Transfer (FRET) efficiency between N-terminally CFP-labeled Kv2.1 and Kv6.4 and C-terminally YFP-labeled KCNE5 (Fig. 7). The FRET efficiency of CFP-Kv6.4 + YFP-Kv2.1 (positive control) and CFP + YFP (negative control) combinations yielded FRET efficiencies of 15.7 ± 1.1% and 1.8 ± 1.0%, respectively. Upon co-expression of KCNE5-YFP with CFP-Kv2.1, we obtained a FRET efficiency of 9.7 ± 0.9% which is lower than the FRET efficiency of the positive control but significantly higher than the FRET efficiency of the negative CFP+YFP combination. This indicated that KCNE5 physically associates with Kv2.1 subunits. Co-expression of KCNE5-YFP with CFP-Kv6.4 yielded a FRET efficiency of 2.4 ± 1.0%. This FRET efficiency was similar to the negative control indicating that KCNE5 does not associate with Kv6.4 subunits alone. These results suggest that KCNE5 subunits modulate the Kv2.1/Kv6.4 heterotetramers via association with Kv2.1 subunits. To investigate this further, we performed FRET experiments co-expressing unlabeled Kv2.1 subunits; an increased FRET efficiency between CFP-Kv6.4 and KCNE5-YFP subunits upon co-expression with unlabeled Kv2.1 would indicate that Kv6.4 and KCNE5 are in each other’s proximity caused by an association with the Kv2.1 subunits into a tripartite complex. Indeed, co-expression of CFP-Kv6.4 with KCNE5-YFP and unlabeled Kv2.1 yielded a FRET efficiency of 6.0 ± 0.9% which was significantly higher than the FRET efficiency obtained with the CFP-Kv6.4 + KCNE5-YFP combination. In addition, this FRET efficiency was similar to the FRET efficiency between CFP-Kv2.1 and KCNE5-YFP upon co-expression with unlabeled Kv6.4 subunits (5.9 ± 1.7%). To ensure that these observed FRET efficiencies were originating from the formed (tripartite) channel complexes (rather than from non-specific interactions caused by the presence of the different tags), we confirmed that the present tags do not affect the biophysical properties of the channels (Suppl. Fig. S6).

## Discussion

Kv2.1 channel activity has been shown crucial for neuronal excitability and neuroprotection (for review see^20^). To accommodate a variety of functions, the Kv2.1 channel diversity is increased through different mechanisms: i) heterotetramerization with modulatory α-subunits of the Kv5, Kv6, Kv8 and Kv9 subfamilies (the so-called silent KvS subunits, for review see^2^), ii) post-translational modifications such as (de)phosphorylation (for review see^21^) and SUMOylation^22^, and iii) association with auxiliary β-subunits such as KChAP^3^, AMIGO^4^ and KCNE proteins (for review, see^8^). Kv2.1 is one of the many channels influenced by KCNE co-assembly and previous studies have indicated a role for KCNE1-3 modulation^5^,^6^. These studies revealed that human KCNE2 and KCNE3 significantly slowed the activation of rat Kv2.1 currents at +60 mV while KCNE1 had a smaller, although still significant, effect^5^,^6^. Our results reveal that human KCNE1 and KCNE3 only slightly slow human Kv2.1 activation at +60 mV (Suppl. Fig. 1). Furthermore, human KCNE2 had no significant effects on human Kv2.1 currents (Suppl. Fig. 1). This discrepancy compared to earlier reports might be explained by the difference in Kv2.1 species. It has been shown that rat Kv2.1 activates faster than human Kv2.1^23^ creating the possibility that the effect of KCNE1-3 subunits on rat Kv2.1 seems larger but that the difference in effect mainly comes from the differences at basal levels. Indeed, the activation kinetics of rat Kv2.1 after modulation by human KCNE1, KCNE2 and KCNE3 subunits are similar to the human Kv2.1 activation kinetics upon co-expression with KCNE1-3 (~15 ms)^5^,^6^.

Co-expression of both KCNE4 and KCNE5 reduced Kv2.1 current densities significantly without affecting the channel gating and inactivation properties (Fig. 1 and Fig. 2, respectively). These results could be explained mainly by modulation of the trafficking and/or targeting of Kv2.1 channels which could occur via several possible mechanisms. For example, KCNE4-5 subunits may suppress the forward trafficking like the demonstrated effect of KCNE1 and KCNE2 on the forward trafficking of Kv1.4, Kv3.3 and Kv3.4^24^. However, we detected KCNE5 subunits in recycling endosomes (Suppl. Fig. S5) opening up for the possibility that KCNE5 reduces the Kv2.1 current density by increasing the endocytosis of Kv2.1 channels as has been suggested for KChIP subunits and Kv4 channels^25^. KCNE4-5 subunits may also decrease the Kv2.1 current density by favoring the localization of Kv2.1 channels into clusters; we detected KCNE5 and Kv2.1 in the same clusters at the plasma membrane (Fig. 6E). It has been demonstrated that Kv2.1 channels that reside within membrane clusters are in a non-conducting state while those outside the clusters are in an conducting state^26^. Another possibility is that KCNE4-5 subunits reduce Kv2.1 current density by enhancing the Kv2.1 forward trafficking (resulting in an increased Kv2.1 surface density) as it has been demonstrated that even the Kv2.1 channels outside the typical Kv2.1 membrane clusters may cease conducting when the Kv2.1 surface current density increases^27^.

It has previously been suggested that different auxiliary β-subunits can associate simultaneously within Kv channel complexes. For example, the simultaneous presence of KChAP and Kvβ1.2 proteins disturbs each other’s effect on Kv1.4, Kv1.5, Kv2.1 and Kv4.3 channels^11^ while KCNE1 (that increases the Kv7.1 current density) and KCNE4 (that decreases the Kv7.1 current density) appear to co-associate with Kv7.1 channels into a “triple subunit” channel complex in which KCNE4 suppresses the KCNE1 effect^12^,^28^. Our data showed that auxiliary β-subunits (i.e. KCNE5) and modulatory α-subunits (i.e. Kv6.4) can associate simultaneously in a tripartite channel complex with Kv2.1 subunits. KCNE5 affected the Kv2.1-containing channels differently in the presence of Kv6.4: KCNE5 modulated the biophysical properties of Kv2.1/Kv6.4 heterotetramers without changing Kv2.1/Kv6.4 current densities (Figs 2, 3, 4). In contrast, KCNE5 reduced Kv2.1 current densities significantly without altering Kv2.1 biophysical properties (Fig. 2). Co-expression of KCNE5 only modified activation, deactivation and inactivation properties of Kv2.1/Kv6.4 heterotetramers significantly (and not those of Kv2.1 homotetramers) which may suggest that the KCNE5 effect requires the presence of the modulatory Kv6.4 α-subunit (or perhaps other KvS α-subunits). This raises the possibility that β-subunits that seemingly have no effect on channel gating and channel inactivation in heterologous expression systems might affect these channel properties *in vivo* when in complex with other (unknown) modulatory α-subunits.

Using FRET experiments, we demonstrated that KCNE5 interacts with Kv2.1 but not with Kv6.4 subunits when co-transfected with either subunit into HEK293 cells. However, FRET occurred between CFP-Kv6.4 and KCNE5-YFP in the presence of unlabeled Kv2.1 (Fig. 7). We envision (at least) three possible mechanisms: i) assembly with Kv2.1 subunits brings Kv6.4 and KCNE subunits together in the same subcellular compartment allowing an interaction between Kv6.4 and KCNE5, ii) conformational changes of Kv6.4 upon co-assembly with Kv2.1 enabling an interaction between Kv6.4 and KCNE5, or iii) Kv6.4 and KCNE5 come in close proximity of each other (without physically interacting with each other) in Kv2.1/Kv6.4/KCNE5 channel complexes resulting in FRET. The latter possibility would implicate that KCNE5 modulates Kv2.1/Kv6.4 biophysical properties via interaction with Kv2.1 subunits.

Kv2.1 channels are ubiquitously expressed and play many diverse roles in both excitable and non-excitable cells. For example, Kv2.1 channels regulate the action potential in hippocampal neurons, especially during high-frequency stimulation^29^, they contribute to both the rapidly activating, slowly inactivating current (I_Ks_) and the non-inactivating, steady-state current (I_ss_) in mouse atrial myocytes^30^ and they play a central role in the regulation of the pancreatic β-cell membrane potential^31^. Compared to ubiquitously expressed Kv2.1 channels, KvS subunits display a more tissue-specific expression creating tissue-specific functions for these Kv2/KvS heterotetramers. Indeed, mutations in Kv8.2 have been associated with an inherited retinal dystrophy^32^ and epilepsy susceptibility^33^ while Kv9.3 subunits play a role in hypoxic pulmonary artery vasoconstriction^34^. Our data demonstrate an association of KCNE5 with Kv2.1 homotetramers and Kv2.1/Kv6.4 heterotetramers. In addition, Kv2.1, Kv6.4 and KCNE5 display an overlapping expressing pattern. For example, both KCNE5 and Kv2.1 can be found in human heart^16^,^28^,^35^,^36^ and Kv2.1, KCNE5 and Kv6.4 are all expressed in human brain^14^,^15^,^16^. This creates the possibility that KCNE5 subunits also affect Kv2.1 homotetramers and Kv2.1/Kv6.4 heterotetramers *in vivo*, resulting in more tissue-specific fine-tuning mechanisms.

## Material and Methods

### Molecular Biology

Human Kv2.1 (GenBank Accession number NM_004975) and human Kv6.4 (NM_172347) in the eGFP-N1 vector (Clontech, Palo Alto, CA, USA) and N-terminally CFP-labeled Kv2.1 and Kv6.4 subunits in the eCFP-C1 vector (Clontech) have been described previously^14^,^17^. Mouse KCNE4 (NM_021342) was amplified from mouse genomic DNA and subcloned in the pBK vector using SmaI. Human KCNE5 (NM_012282) was amplified from human genomic DNA and subcloned into either the pBK vector using SalI and BamHI or into the pXOOM vector using BamHI and EcoRI. C-terminally YFP-labeled KCNE5 was obtained by subcloning the KCNE5 cDNA in the peYFP-N1 (Clontech) vector. HA-Kv6.4 and HA-KCNE5 constructs were generated by introducing the HA epitope (YPYDVPDYA) into the extracellular S1-S2 loop and the extracellular N-terminus, respectively, by PCR amplification using the QuikChange^TM^ Site-Directed Mutagenesis kit (Stratagene, La Jolla, CA, USA) and mutant primers. All PCR-generated inserts were sequenced to confirm sequence integrity and in-frame clonings.

### Cell culture and transfection

HEK293 cells were cultured in Modified Eagle’s medium (Invitrogen, San Diego, CA, USA) supplemented with 10% fetal bovine serum, 1% non-essential amino acids and 1% penicillin-streptomycin at 37 °C and 5% CO_2_. Cells were co-transfected with the subunit cDNAs and GFP as a transfection marker using Lipofectamine2000 (Invitrogen, San Diego, CA, USA). The transfections were performed in 60 mm cell culture dishes filled with 4 ml culture medium for the electrophysiological and Fluorescence Resonance Energy Transfer (FRET) experiments and in 35 mm cell culture dishes filled with 2 ml culture medium for the immunocytochemical experiments. 16–24 h after transfection, cells were dissociated with trypsin and used for electrophysiological analysis within 5 h or fixated on coverslips using 4% cold paraformaldehyde for 10 min for immunocytochemistry.

### Electrophysiology

Electrophysiological recordings were performed as previously described^19^. Briefly, whole cell current recordings were performed at 20–22 °C using an Axopatch-200B amplifier (Axon Instruments, Union City, CA, USA) connected to a Digidata 1440 data acquisition system (Axon Instruments) and were low-pass filtered and sampled at 1–10 kHz. Command voltages and data storage were controlled with the pClamp10 software (Axon Instruments). Patch pipettes with a resistance of 1.5–2.5 MΩ were filled with an intracellular solution containing (in mM): 110 KCl, 5 K_4_BAPTA, 5 K_2_ATP, 1 MgCl_2_ and 10 HEPES, pH adjusted to 7.2 with KOH. Cells were continuously perfused with an extracellular solution containing (in mM): 145 NaCl, 4 KCl, 1.8 CaCl_2_, 1 MgCl_2_, 10 HEPES, 10 glucose, pH adjusted to 7.35 with NaOH. Cells were excluded from analysis if voltage errors at the highest potentials used (i.e. 60 mV and 70 mV to determine the inactivation and (de)activation properties, respectively) did exceed 5 mV after series resistance compensation.

### Pulse protocols and data analysis

The Boltzmann equation: y = 1/{1 + exp[−(V − V_1/2_)/k]} with V the voltage applied, V_1/2_ the voltage at which 50% of the channels are (in)activated and k the slope factor was fitted to the voltage-dependence of activation and inactivation. Single or double exponential function were fitted to the (de)activation kinetics. The Hill equation: 1 - y = 1/(1-(IC_50_/[D]^n^_H_) with IC_50_ the concentration that generates 50% current inhibition, [D] the drug concentration and n_H_ the Hill coefficient was fitted to the concentration-effect curves. Statistical analysis was performed using Student’s t test or Mann-Whitney U Rank Sum test.

### Fluorescence Resonance Energy Transfer (FRET)

FRET experiments were performed as previously described^17^. Briefly, HEK293 cells were cultured on coverslips and co-transfected with YFP (acceptor fluorophore) and CFP (donor fluorophore) labeled subunits. 48 h after transfection, FRET was determined using a Zeiss CLSM 510 microscope equipped with an argon laser to visualize and bleach CFP (with excitation at 458 nm) and to bleach YFP (with excitation at 514 nm). FRET efficiencies were determined with following equation: FRET efficiency = (1-(f_*DA*_-f_*background*_)/(f_*D*_-f_*background*_))x(1/paired*DA*) in which f_*DA*_ and f_*D*_ represent the donor fluorescence in the presence and absence of the acceptor, respectively, f_*background*_ the background fluorescence and paired*DA* the fraction of paired donor and acceptor fluorophores. F_*DA*_, f_*D*_ and f_*background*_ were obtained by recording the CFP emission in the 464–490 nm bandwidth without YFP bleaching, after YFP bleaching by a 30 s full power excitation at 514 nm and after CFP bleaching by a 30 s full power excitation at 458 nm, respectively. Paired*DA* was assumed to be 1 which was pursued by i) transfecting the CFP- and YFP-tagged subunits in a 1:2 cDNA ratio and ii) maintaining a YFP/CFP fluorescence intensity ratio <1 that was determined from the intensities in the YFP and CFP bandwidth (532–554 nm and 464–490 nm, respectively) before YFP and CFP bleaching. A paired*DA* <1 will result in an underestimation of the FRET efficiency and cells with an YFP/CFP intensity ratio <1 were excluded from FRET efficiency analysis. Because neither CFP nor YFP carried the mutation A206K implicating that they have the weak tendency to dimerize, the FRET efficiency might be slightly under or overestimated.

### Immunocytochemistry

Fixated cells first underwent a combined permeabilization and blocking step for 30 min at room temperature using 0.1% Triton X-100 and 0.2% fish skin gelatin in 1xPhosphate Buffered Saline (PBS) (immunobuffer). Secondly, primary antibodies diluted in immunobuffer were applied for 1 h followed by 3 washing steps, 5 min each, using 0.1% Triton X-100 in PBS. Alexa-Fluor conjugated secondary antibodies (Invitrogen) were diluted in immunobuffer and applied for 45 min before coverslips were mounted in Prolong Gold Antifade reagent (Invitrogen).

### Confocal microscopy and imaging

All images were acquired using the laser scanning confocal microscope Zeiss LSM780 equipped with a 63x/NA = 1.40 oil objective. The pinhole diameter was set between 0.7–1.0 μm equaling 1 airy unit. Images were obtained using sequential scanning separating the individual channels and noise was reduced employing line averaging. Finally, images were treated with ZEN 2011 edition and Adobe Illustrator and Photoshop CS6.

### Primary antibodies

Rabbit polyclonal anti-KCNE5 (1:100, in-house), mouse monoclonal anti-Kv2.1 (1:100, clone K39/25, NeuroMab, UC Davis/NIH NeuroMab Facility, Davis, CA, USA), and rat monoclonal anti-HA (1:50, clone 3F10, Roche, Indianapolis, USA) were used to detect KCNE5, Kv2.1, HA-Kv6.4, or HA-KCNE5, respectively. Alexa-Fluor 647-conjugated and Rhodamine-conjugated phalloidin (1:200, Invitrogen) were used as plasma membrane markers in HEK293 cells. Mouse monoclonal Early Endosome Antigen 1 (EEA1) (1:100, clone 14, BD Transduction Laboratories, New Jersey, USA), mouse monoclonal Golgin-97 (1:50, clone CDF4, Invitrogen), mouse monoclonal mannose-6-phosphate receptor (M6PR) (1:100, clone 2G11, Abcam, Cambridge, UK) and mouse monoclonal Transferrin receptor (1:100, clone H68.4, Invitrogen) were used to detect early endosomes, the Golgi Apparatus, late endosomes/prelysosomes, or recycling endosomes, respectively.

## Additional Information

**How to cite this article**: David, J.-P. *et al.* Auxiliary KCNE subunits modulate both homotetrameric Kv2.1 and heterotetrameric Kv2.1/Kv6.4 channels. *Sci. Rep.* **5**, 12813; doi: 10.1038/srep12813 (2015).

## Supplementary Material

Supplementary Information

## Figures and Tables

**Table 1:** Biophysical Kv2.1 and Kv2.1/Kv6.4 properties in the absence and presence of KCNE4 and KCNE5.

Activation							
	*V*_*1/2*_	*k*	*n*		*V*_*1/2*_	*k*	*n*
Kv2.1	2.23 ± 2.23	8.86 ± 0.73	6	+ Kv6.4	−3.05 ± 2.71	15.67 ± 13.4	5
+ KCNE4	2.36 ± 3.61	9.66 ± 1.04	5	+ KCNE4	−6.5 ± 2.06	18.80 ± 1.64	7
+ KCNE5	−2.09 ± 1.55	7.98 ± 0.91	7	+ KCNE5	−7.48 ± 3.32	16.62 ± 1.90	8
Inactivation							
	*V*_*1/2*_	*k*	*n*		*V*_*1/2*_	*k*	*n*
Kv2.1	−21.21 ± 2.21	6.00 ± 0.53	5	+ Kv6.4	−57.87 ± 2.34	11.10 ± 1.37	10
+ KCNE4	−22.87 ± 0.84	6.27 ± 0.44	5	+ KCNE4	−57.88 ± 1.80	10.02 ± 1.55	6
+ KCNE5	−21.56 ± 2.19	4.69 ± 0.83	5	+ KCNE5	−58.18 ± 1.57	**6.60** ± **0.35**	16
Activation time constants at 20 mV							
	*Tau*		*n*		*Tau1*	*Tau2*	*n*
Kv2.1	18.51 ± 1.81		5	+ Kv6.4	32.96 ± 3.24	n.a.	5
+ KCNE4	20.23 ± 1.23		5	+ KCNE4	26.31 ± 4.17	n.a.	7
+ KCNE5	21.10 ± 1.58		5	+ KCNE5	**20.16** ± **1.45**	69.28 ± 2.76	8
Activation time constants at 60 mV							
	*Tau*		*n*		*Tau1*	*Tau2*	*n*
Kv2.1	11.12 ± 0.69		5	+ Kv6.4	18.14 ± 1.97	39.77 ± 3.83	5
+ KCNE4	11.62 ± 0.59		5	+ KCNE4	**11.67** ± **1.06**	43.96 ± 6.64	7
+ KCNE5	11.71 ± 1.13		5	+ KCNE5	**8.94** ± **0.49**	26.85 ± 3.67	8
Deactivation time constants at −30 mV							
	*Tau1*	*Tau2*	*n*		*Tau1*	*Tau2*	*n*
Kv2.1	16.60 ± 1.22	92.92 ± 14.11	4	+ Kv6.4	18.68 ± 1.07	111.76 ± 19.67	3
+ KCNE4	18.01 ± 1.64	90.16 ± 18.92	3	+ KCNE4	18.34 ± 2.16	116.48 ± 32.25	4
+ KCNE5	20.03 ± 2.18	86.42 ± 7.24	4	+ KCNE5	**31.40** ± **2.37**	179 ± 27.56	5

Values are given as mean ± S.E.M. Values in bold are significantly different (p < 0.05) compared to controls. V_1/2_, midpoint of activation or inactivation (in mV); k, slope factor; Tau, time constant (in ms); n, number of cells; n.a., not applicable.
