# Supplementary material for: Auxiliary KCNE subunits modulate both homotetrameric Kv2.1 and heterotetrameric Kv2.1/Kv6.4 channels
Source: Sci Rep. 2015 Aug 5;5:12813. doi: 10.1038/srep12813 (PMC4525287; doi:10.1038/srep12813)
Supplement: Supplementary Information [file srep12813-s1.pdf]

Supplemental material

Auxiliary KCNE subunits modulate both homotetrameric Kv2.1 and heterotetrameric Kv2.1/Kv6.4 channels

Jens-Peter David [1], Jeroen I. Stas [2], Nicole Schmitt [1], Elke Bocksteins [2]

[1] Danish National Research Foundation Centre for Cardiac Arrhythmia and Department for Biomedical Sciences, Faculty of Health and Medical Sciences, University of Copenhagen, Copenhagen, Denmark
[2] Laboratory for Molecular Biophysics, Physiology and Pharmacology, Department for Biomedical Sciences, University of Antwerp, Antwerp, Belgium

Table of Contents

Supplemental material and methods 2
- Molecular Biology 2
- Generation of KCNE5 antibody 2
- Immunoblotting 2

Supplemental Tables 3
- Supplemental Table S1: Overview of biophysical Kv2.1 and Kv2.1/Kv6.4 properties in the absence and presence of KCNE1, KCNE2 and KCNE3 3
- Supplemental Table S2: Overview of biophysical properties of (CFP-labeled) Kv2.1 and (HA- or CFP-labeled) Kv2.1/Kv6.4 in the presence of (YFP-labeled) KCNE5 4

Supplemental Figures 5
- Supplemental Figure S1: Biophysical properties of Kv2.1 homotetramers alone or upon co-expression with KCNE1, KCNE2 and KCNE3 5
- Supplemental Figure S2: Biophysical properties of Kv2.1/Kv6.4 heterotetramers alone or upon co-expression with KCNE1, KCNE2, and KCNE3 6
- Supplemental Figure S3: Characterization of KCNE5 antibody 7
- Supplemental Figure S4: Surface staining of KCNE5 subunits containing an extracellular positioned HA-tag 8
- Supplemental Figure S5: Co-stainings of KCNE5 together with various compartmental markers 9
- Supplemental Figure S6: Biophysical properties of (CFP-labeled) Kv2.1 and (HA- or CFP-labeled) Kv2.1/Kv6.4 in the presence of (YFP-labeled) KCNE5. 10

Supplemental References 11

Supplemental material and methods

Molecular Biology

Human KCNE1 (GenBank Accession number NM_000219.5), human KCNE2 (NM_172201), and human KCNE3 (NM_005472) were amplified from human genomic DNA and subcloned in the pBK vector using SmaI restriction enzyme digest. Plasmid pDsRed2-ER (DsRed-ER) from Clontech was used for fluorescent labeling of the endoplasmic reticulum (ER).

Generation of KCNE5 antibody

A polyclonal serum was raised against the human KCNE5 protein (NM_012282) using the sequence RQLASEGLPALQGAERV located at the distal C-terminus of the KCNE5 protein (PolyPeptide Laboratories France SAS, Strasbourg, France). The antibody KCNE5 was affinity-purified as described in detail previously [1]. The antibody specificity was verified by incubation with pre-immune serum and by blocking experiments with the peptide the antibody was raised against.

Immunoblotting

For preparation of lysates, HEK293 cells were grown to confluency and washed with ice-cold phosphate-buffered saline (PBS, in mM: NaCl 136; KCl 2.5; KH_2PO_4 1.5; $NaHPO_4$ 6.5; pH 7.4). The cells were suspended in modified RIPA buffer (in mM: Tris/HCl 50; NaCl 150; NaF 1; Na_3VO_4 1; 0,5 % Triton X-100, and protease inhibitors, pH 7,4) and incubated for 3 hours at 4℃ with gentle shaking. Lysates were centrifuged at 10,000g for 15 min at 4℃, supernatants were collected, and protein concentration was determined by Bradford assay. The lysates were not subjected to heat treatment prior to Western Blot analysis to avoid aggregation and degradation. Non-heat treated lysates (25 µg/lane) were separated on 7,5 % SDS-PAGE polyacrylamide gels using the Bio-Rad Laboratories minigel system (Hercules, CA, USA). Proteins were transferred onto a nitrocellulose transfer membrane in 25 mM Tris base, 200 mM glycine, 20 % methanol using a mini transblot (Bio-Rad). After transfer, the membranes were incubated for 1 hour at room temperature in blocking buffer (PBS containing 4% low-fat milk powder). The membrane was incubated overnight at 4ºC in blocking buffer containing rabbit anti-KCNE5 in 1:1000 dilution. After washing, bound antibody was revealed with HRP-conjugated donkey anti-rabbit antibody (1:10000, Jackson Immunosearch Laboratories, Westgrove, PA, USA) in blocking buffer for 45 min and detected by enhanced chemiluminescence (ECL). Immunoblots were exposed on hyperfilm ECL (Amersham Biosciences).

Supplemental Tables

Supplemental Table S1: Overview of biophysical Kv2.1 and Kv2.1/Kv6.4 properties in the absence and presence of KCNE1, KCNE2 and KCNE3.

Activation

	$V_{1/2}$	*k*	*n*		*$V_{1/2}$*	*k*	*n*
Kv2.1	2.23 ± 2.23	8.86 ± 0.73	6	+ Kv6.4	-3.05 ± 2.71	15.67 ± 13.4	5
+ KCNE1	3.23 ± 0.36	11.94 ± 2.31	5	+ KCNE1	-8.55 ± 2.32	16.36 ± 3.24	5
+ KCNE2	0.47 ± 1.35	7.74 ± 0.64	8	+ KCNE2	-7.15 ± 2.07	15.86 ± 1.91	6
+ KCNE3	2.61 ± 2.14	11.47 ± 1.05	6	+ KCNE3	-8.10 ± 2.35	16.33 ± 1.96	7

Inactivation

	$V_{1/2}$	*k*	*n*		*$V_{1/2}$*	*k*	*n*
Kv2.1	-21.21 ± 2.21	6.00 ± 0.53	5	+ Kv6.4	-57.87 ± 2.34	11.10 ± 1.37	10
+ KCNE1	-26.56 ± 1.58	6.29 ± 0.60	5	+ KCNE1	-55.48 ± 5.14	10.68 ± 1.65	5
+ KCNE2	-24.60 ± 3.79	5.15 ± 0.58	5	+ KCNE2	-58.22 ± 1.59	9.06 ± 0.90	5
+ KCNE3	-25.33 ± 2.92	5.81 ± 0.34	6	+ KCNE3	-57.14 ± 2.39	9.55 ± 1.16	5

Activation time constants

	Tau at +60 mV	*n*		*Tau1 at +60 mV*	*Tau2 at +60 mV*	*n*
Kv2.1	11.12 ± 0.69	5	+ Kv6.4	18.14 ± 1.97	39.77 ± 3.83	5
+ KCNE1	**14.16 ± 1.21**	5	+ KCNE1	13.50 ± 1.71	34.99 ± 8.76	5
+ KCNE2	11.20 ± 0.46	5	+ KCNE2	**11.12 ± 1.23**	37.11 ± 5.89	6
+ KCNE3	**13.93 ± 0.97**	6	+ KCNE3	13.87 ± 1.44	31.90 ± 8.83	7

Deactivation time constants

	Tau1 at -30 mV	*Tau2 at -30 mV*	*n*		*Tau1 at -30 mV*	*Tau2 at -30 mV*	*n*
Kv2.1	16.60 ± 1.22	92.92 ± 14.11	4	+ Kv6.4	18.68 ± 1.07	111.76 ± 19.67	3
+ KCNE1	18.69 ± 1.32	64.88 ± 12.85	3	+ KCNE1	20.06 ± 4.7	138.34 ± 33.15	3
+ KCNE2	15.01 ± 2.77	81.17 ± 19.50	2	+ KCNE2	23.43 ± 1.62	108.2 ± 28.18	3
+ KCNE3	17.55 ± 0.92	67.53 ± 5.41	6	+ KCNE3	15.50 ± 0.34	98.76 ± 17.74	4

Values are given as mean ± S.E.M. and values in bold are significantly different ($p < 0.05$) compared to control values. $V_{1/2}$, midpoints of activation or inactivation (in mV); k, slope factor; Tau, time constants (in ms); n, number of cells.

Supplemental Table S2: Overview of biophysical properties of (CFP-labeled) Kv2.1 and (HA- or CFP-labeled) Kv2.1/Kv6.4 in the presence of (YFP-labeled) KCNE5.

	CFP-Kv2.1 + KCNE5-YFP	Kv2.1 + Kv6.4-HA + KCNE5	Kv2.1 + CFP-Kv6.4 + KCNE5-YFP	CFP-Kv2.1 + Kv6.4 + KCNE5-YFP
Activation				
$V_{1/2}$ (mV)	-1.4 ± 1.2	-4.3 ± 1.0	-7.2 ± 3.4	-4.2 ± 2.6
k	7.1 ± 0.5	13.0 ± 1.7	16.6 ± 1.6	17.3 ± 1.9
Tau1 at 20 mV	25.91 ± 0.67	24.03 ± 0.57	22.82 ± 2.56	26.57 ± 4.35
Tau1 at 60 mV	13.59 ± 0.88	10.42 ± 1.16	9.83 ± 1.18	9.66 ± 1.06
n	6	7	7	5
Inactivation				
$V_{1/2}$ (mV)	-20.3 ± 1.1	-63.5 ± 2.8	-58.2 ± 2.9	-58.3 ± 1.7
k	4.9 ± 0.1	7.4 ± 0.4	7.3 ± 1.0	6.3 ± 0.2
n	4	3	7	6
Deactivation				
Tau1 at -30 mV	14.65 ± 0.95	25.90 ± 2.95	30.45 ± 5.10	28.90 ± 1.16
Tau2 at -30 mV	73.11 ± 0.18	188.89 ± 29.91	134.05 ± 19.14	148.81 ± 67.45
n	3	3	3	3

Values are given as mean ± S.E.M. $V_{1/2}$, midpoints of activation or inactivation (in mV); k, slope factor; Tau, time constants (in ms); n, number of cells.

Supplemental Figures

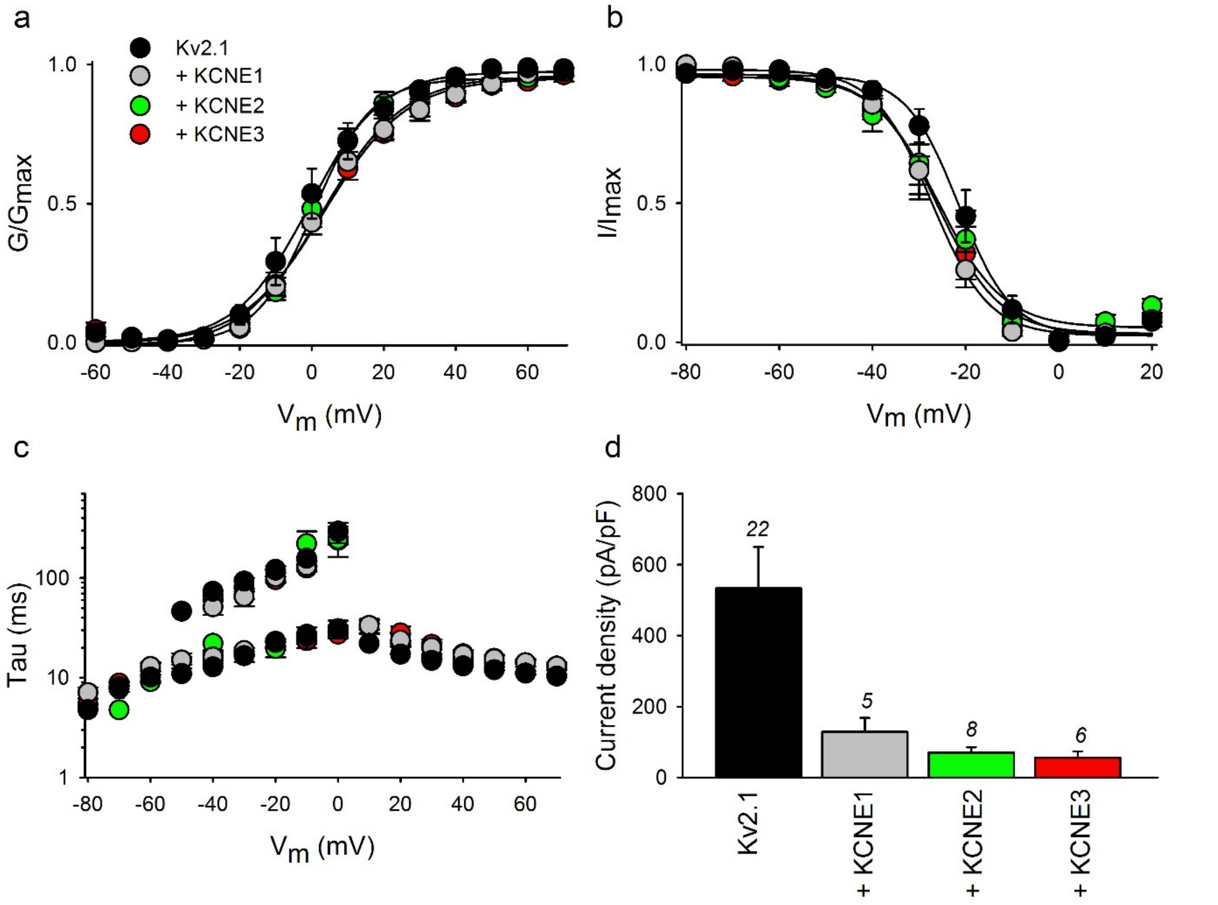

Supplemental Figure S1: Biophysical properties of Kv2.1 homotetramers alone or upon co-expression with KCNE1, KCNE2 and KCNE3. **A.** Voltage-dependence of activation of Kv2.1 in the absence and presence of KCNE1, KCNE2, and KCNE3. The voltage-dependence of activation was determined by plotting the normalized tail currents at -35 mV as a function of the prepulse potential (ranging from -70 mV to +70 mV in 10-mV increments). Solid lines represent the Boltzmann fit. KCNE1-3 subunits did not modulate the Kv2.1 voltage-dependence of activation. **B.** Voltage-dependence of Kv2.1 inactivation in the absence or presence of KCNE1-3 subunits. The voltage-dependence of inactivation was determined by plotting the normalized current amplitude at +60 mV as a function of the 5-s prepulse potential. KCNE1, KCNE2, and KCNE3 did not alter the voltage-dependence of inactivation of Kv2.1 homotetramers significantly. **C.** Kv2.1 activation and deactivation kinetics in the absence or presence of KCNE1, KCNE2, and KCNE3. The activation and deactivation kinetics are derived from a single or double exponential fit of the raw current recordings. KCNE1-3 did not alter Kv2.1 deactivation kinetics but KCNE1 and KCNE3 slightly modified the Kv2.1 activation kinetics. **D.** Current densities obtained at 0 mV after co-expression of 250 ng Kv2.1 with 1 µg CFP (black), 1 µg KCNE1 (gray), 1 µg KCNE2 (green) or 1 µg KCNE3 (red). Co-expression of KCNE1-3 with Kv2.1 resulted in a significant reduction of the Kv2.1 current density ($p<0.05$); n=number of cells.

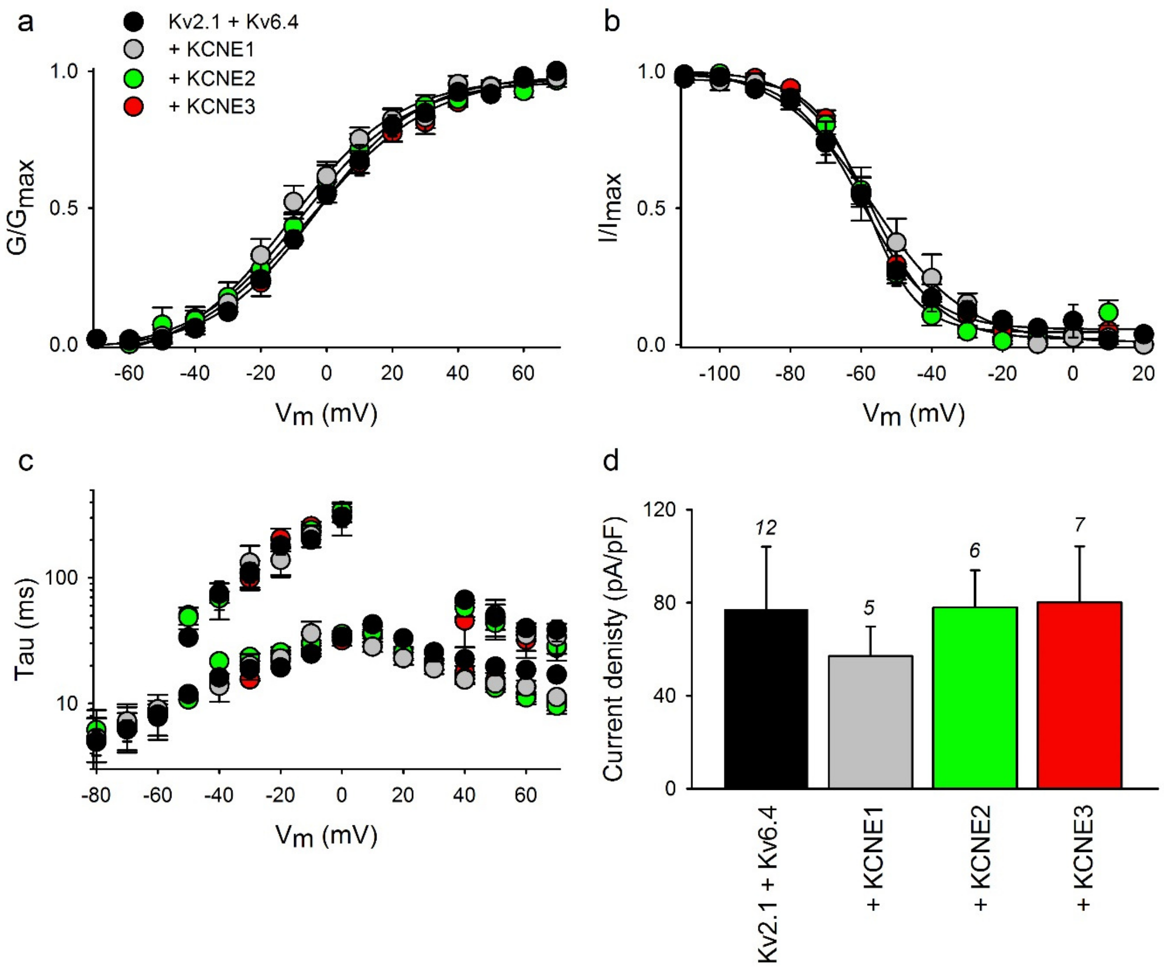

Supplemental Figure S2: Biophysical properties of Kv2.1/Kv6.4 heterotetramers alone or upon co-expression with KCNE1, KCNE2, and KCNE3. **A.** Voltage-dependence of activation of Kv2.1/Kv6.4 heterotetramers in the absence or presence of KCNE1-3 subunits. The activation curves were determined as described in Suppl. Fig. 1A. KCNE1-3 did not alter the voltage-dependence of activation of Kv2.1/Kv6.4 homotetramers significantly. **B.** Voltage-dependence of Kv2.1/Kv6.4 inactivation in the absence or presence of KCNE1, KCNE2, or KCNE3. The inactivation curve was determined as described in Suppl. Fig. 1B. KCNE1, KCNE2 or KCNE3 did not modulate the Kv2.1/Kv6.4 voltage-dependence of inactivation. **C.** Activation and deactivation kinetics of Kv2.1/Kv6.4 in the absence or presence of KCNE1-3 obtained like for Kv2.1 in Suppl. Fig. 1C. KCNE1-3 did not alter the Kv2.1/Kv6.4 kinetics but KCNE2 slightly modified Kv2.1/Kv6.4 activation kinetics. **D.** Current densities obtained at 0 mV after co-expression of 0.5 µg Kv2.1 and 5 µg Kv6.4 with 1 µg CFP (black), 1 µg KCNE1 (gray), 1 µg KCNE2 (green) or 1 µg KCNE3 (red). KCNE1-3 subunits did not affect the Kv2.1/Kv6.4 current density significantly ($p<0.05$); n=number of cells.

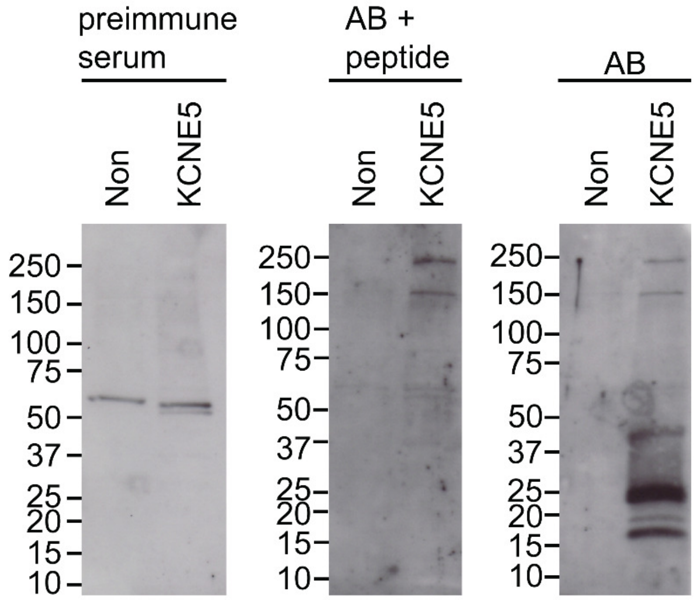

Supplemental Figure S3: Characterization of KCNE5 antibody. We tested the antibody specificity by performing Western blots on whole cell lysates from non-transfected (Non) or KCNE5 transfected (KCNE5) HEK293 cells. Since the signals on the Western blots were not present when the gel was stained with preimmune serum (left panel) or antibody preincubated with the antigenic peptide (middle), we considered the antibody to be specific.

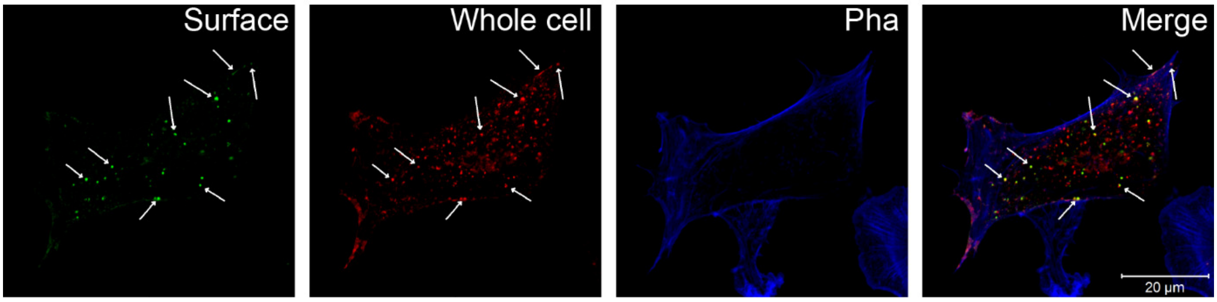

Supplemental Figure S4: Surface staining of KCNE5 subunits containing an extracellular positioned HA-tag. HEK293 cells singly expressing KCNE5 subunits with a HA-tag at the extracellular distal N-terminus were stained with a specific HA-tag antibody without permeabilizing the cell membrane (surface staining, green) and hereby detecting KCNE5 subunits at the plasma membrane. Afterwards, the cells were permeabilized and the whole population of KCNE5 subunits was detected using a KCNE5 specific antibody (whole cell staining, red). Arrows are pointing at examples of KCNE5 clusters at the membrane. Vesicles only positive in the whole cell staining indicate that KCNE5 also locates in some intracellular compartments. Phalloidin (Pha) was used as a membrane marker. The merged picture is shown in the right column.

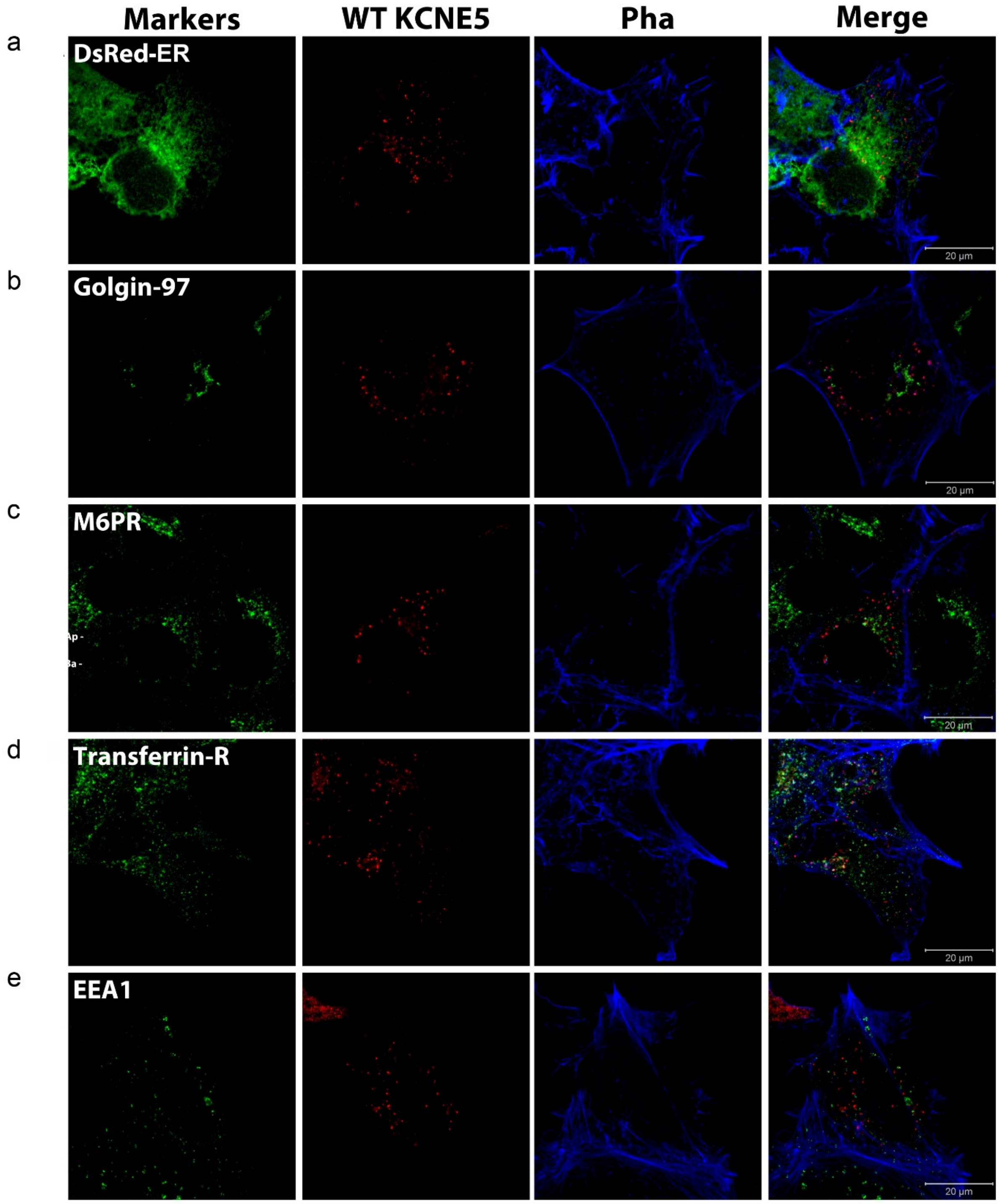

Supplemental Figure S5: Co-stainings of KCNE5 together with various compartmental markers. KCNE5 was transfected into HEK293 cells and detected using a KCNE5 specific antibody (red). **A.** DsRed-ER was used to indicate the ER (green). The KCNE5 staining did not overlap with this ER marker, indicating that KCNE5 is not retained in the ER. **B-E.** KCNE5 containing vesicles are not part of the Golgi apparatus (Golgin-97), late endosomes/prelysosomes (mannose-6-phosphate receptor, M6PR), or early endosomes (Early Endosome Antigen 1, EEA1). Yet, a few of the KCNE5 positive vesicles did seem to overlap with some of the recycling endosomes (Transferrin-R). Phalloidin (Pha) was used as a membrane marker. Images were acquired using laser confocal microscopy and merged in the right column.

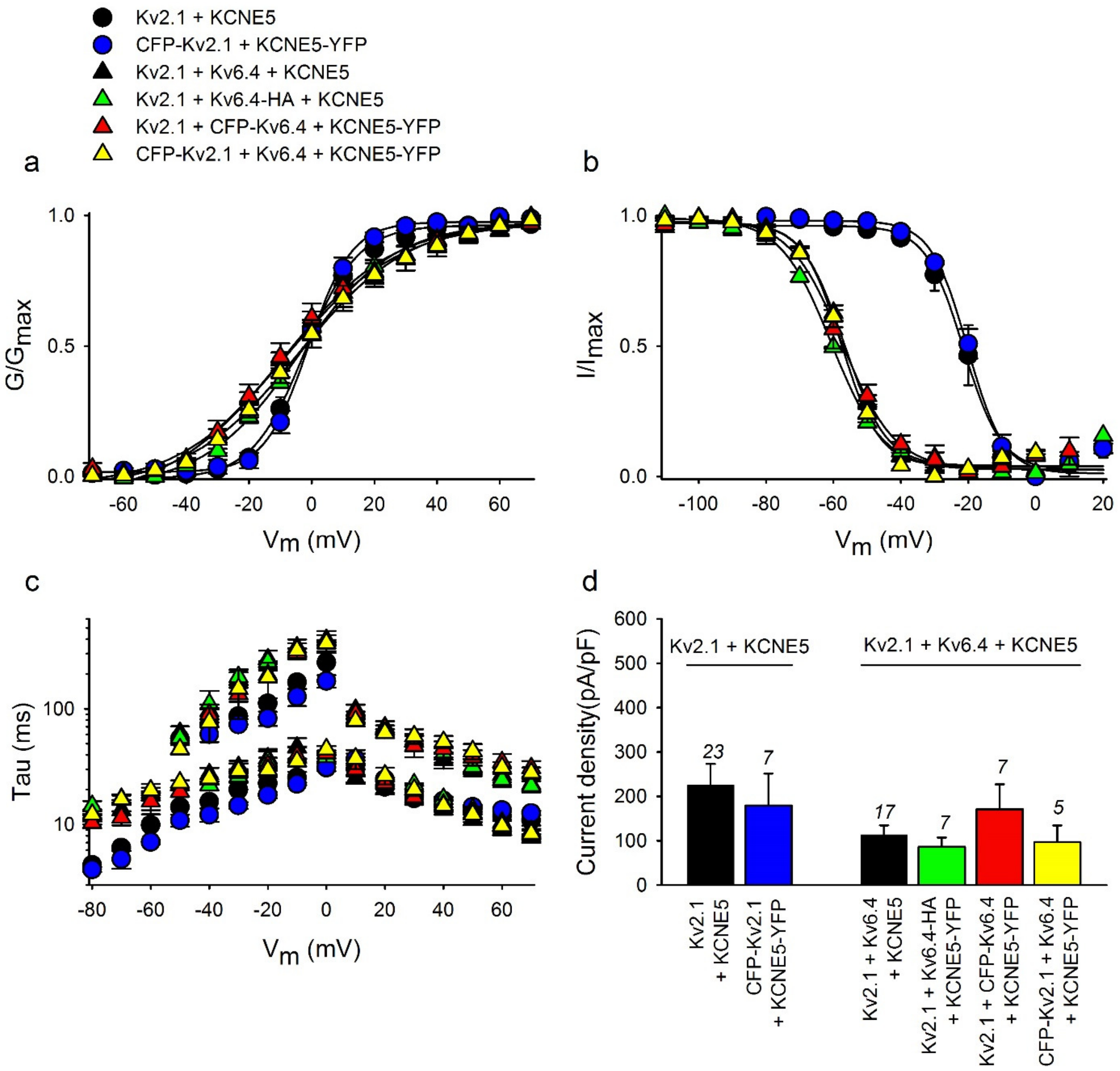

Supplemental Figure S6: Biophysical properties of (CFP-labeled) Kv2.1 and (HA- or CFP-labeled) Kv2.1/Kv6.4 in the presence of (YFP-labeled) KCNE5. Voltage-dependence of activation (**A**), voltage-dependence of inactivation (**B**), activation and deactivation kinetics (**C**) and current density (**D**) of CFP-Kv2.1 + KCNE5-YFP (blue), Kv2.1 + Kv6.4-HA + KCNE5 (green), Kv2.1 + CFP-Kv6.4 + KCNE5-YFP (red) and CFP-Kv2.1 + Kv6.4 + KCNE5-YFP (yellow). For comparison, the relevant properties of the unlabeled Kv2.1/KCNE5 and unlabeled Kv2.1/Kv6.4/KCNE5 channel complexes are shown in black. The voltage-dependence of activation and of inactivation, (de)activation kinetics and current density of each combination were determined as in Suppl. Fig. S1A-D, respectively. Numbers in panel D represent the number of analyzed cells. Note that the presence of one or more labeled subunits within a channel complex did not affect the biophysical properties of that (tripartite) channel complex compared to the representative unlabeled channel complexes.

Supplemental References

[1] H. G. Knaus, *et al.*, "Characterization of tissue-expressed alpha subunits of the high conductance Ca(2+)-activated K+ channel," J. Biol. Chem. **270**(38), 22434 (1995).
